# Flavonoids as Novel Efflux Pump Inhibitors and Antimicrobials Against Both Environmental and Pathogenic Intracellular Mycobacterial Species

**DOI:** 10.3390/molecules25030734

**Published:** 2020-02-07

**Authors:** Julia Solnier, Liam Martin, Sanjib Bhakta, Franz Bucar

**Affiliations:** 1 Institute of Pharmaceutical Sciences, Department of Pharmacognosy, University of Graz, 8010 Graz, Austria; julia.solnier@uni-graz.at; 2 Institute of Structural and Molecular Biology, Department of Biological Sciences, Birkbeck, University of London, London WC1E 7HX, UK; liam.tom.martin@gmail.com (L.M.); s.bhakta@bbk.ac.uk (S.B.)

**Keywords:** flavonoids, skullcapflavone II, nobiletin, efflux pumps, efflux pump inhibitors, mycobacteria

## Abstract

Therapeutic treatment options for opportunistic non-tuberculous mycobacterial (NTM) infection and/or serious mycobacterial infections such as tuberculosis (TB) and leprosy are limited due to the spread of antimicrobial resistance mechanism. Plant-derived natural compounds as prospective efflux pump inhibitors may present a promising adjunct to conventional chemotherapy by enhancing mycobacterial susceptibility to antibiotics. This study served to evaluate the antimicrobial and resistance-modifying profile of a range of plant-derived flavonoids against the mycobacterial model strains: *M. smegmatis*, *M. aurum*, and *M. bovis* BCG. The minimum inhibitory concentrations (MICs) of the compounds against the mycobacterial strains were determined using both agar dilution and broth dilution assays, while their efflux inhibitory activity was investigated via an ethidium bromide-based fluorometric assay. All compounds were screened for their synergistic effects with ethidium bromide (EtBr) and rifampicin (RIF) against *M. smegmatis*. Skullcapflavone II (5,2′-dihydroxy-6,7,8,6′-tetramethoxyflavone, **1**) exerted potent antimicrobial activity against *M. aurum* and *M. bovis* BCG and considerably increased the susceptibility of *M. smegmatis* to EtBr and RIF. Nobiletin (5,6,7,8,3′,4′-hexamethoxyflavone, **2**) was determined to be the most potent efflux-inhibitor in *M. aurum* and *M. smegmatis*. However, a connection between strong modulatory and putative efflux activity of the compounds could not be observed. Nevertheless, the results highlight two polymethoxyflavones, skullcapflavone II and nobiletin, with potent antimycobacterial and antibiotic resistance modulating activities as valuable adjuvants in anti-mycobacterial therapies.

## 1. Introduction

The rapid global expansion of multidrug-resistant (MDR) and extensively drug-resistant (XDR) bacteria reflects the urgent need of a novel course in antibiotic therapy to tackle global infectious diseases such as tuberculosis (TB) [1].

According to the most recent Global Tuberculosis Report from the World Health Organization (WHO), TB remains one of the top 10 causes of death among infectious diseases. Due to the rising number of MDR strains, especially in mycobacteria including the *Mycobacterium tuberculosis* complex, as well as fast growing non-tuberculous strains, antimicrobial resistance has become a critical global health concern [2].

The *Mycobacterium bovis* bacillus Calmette–Guérin (BCG) strain is the most frequently used live attenuated vaccine against tuberculosis disease. The BCG strain was originally derived after several subcultures from its virulent progenitor *Mycobacterium bovis (M. bovis)*, which triggers TB infections in animal species, predominantly cattle [3,4]. Beside the pathogenic mycobacteria, there are numerous opportunistic species occurring as saprophytes and commensals in the environment. These fast-growing and non-obligatory pathogenic organisms are categorized as non-tuberculous mycobacteria (NTM) that readily cause opportunistic infections in immunocompromised patients [5,6]. Among them, *Mycobacterium smegmatis* and *Mycobacterium aurum* regularly serve as low-pathogenic and rapidly growing surrogate models in antitubercular drug screening for antitubercular drugs [7,8]. Due to their genomic similarities and the correlation between their antibiotic susceptibility profile and that of *M. tuberculosis*, the use of model strains [9] including *M. smegmatis*, *M. aurum*, and *M. bovis* BCG accelerates the discovery of new antitubercular drugs, while lowering the risk to researchers, and allowing for screening of compounds in labs that lack Category 3 bio-safety facilities [6,10].

A distinctive feature of mycobacteria is their highly hydrophobic cell envelope and the prevalence of multidrug efflux pumps (EPs). Putative drug efflux genes and homologous pumps can be found in *M. smegmatis, M. aurum, M. bovis,* and *M. tuberculosis* [11,12,13]. These EPs represent one of many important resistance mechanisms developed by bacteria to survive in the presence of chemotherapeutic drugs [14]. By expelling toxic substrates from the bacterial cell, these transmembrane proteins operate as effective tools in order to prevent the intracellular accumulation of antimicrobial drugs [15,16]. Consequently, the inhibition of efflux pumps may be an effective strategy to assist in the fight against rising antibiotic resistance while initiating a new procedure in drug-therapy [17,18].

**Figure 1:**
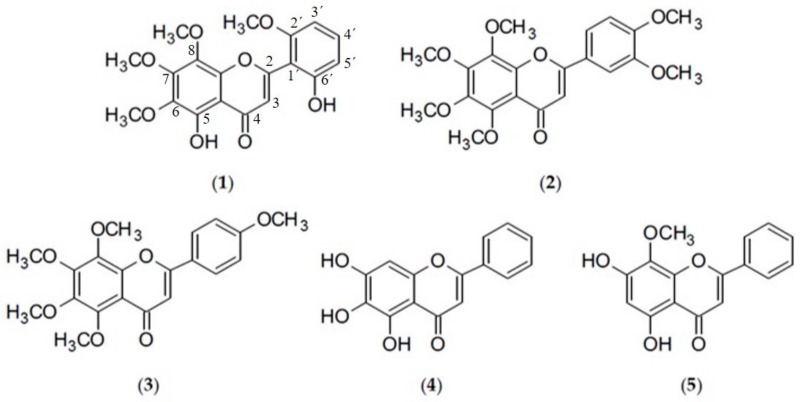
Chemical structures of skullcapflavone II (**1**), nobiletin (**2**), tangeretin (**3**), baicalein (**4**), and wogonin (**5**).

Despite the fact that a number of challenges has to be overcome, like the risk of resistance development when mycobacteria are exposed to subinhibitory concentrations of potential efflux pump inhibitors (EPI), similar pharmacokinetics of adjuvant and antitubercular drugs or selectivity of EPI for bacterial efflux pumps rather than eukaryotic transporters, the inclusion of an EPI as part of a therapeutic regimen could revive the therapeutic efficacy of the fading antibiotic arsenal [10]. However, to date, no efflux pump inhibitor has been clinically approved [19]. Recently, interest has been growing in the identification of new efflux pump inhibitors from natural sources [20], including flavonoids. A number of flavonoids have been shown to increase susceptibility of NTM to isoniazid, the flavonol myricetin being the most active [21]. Further, the isoflavone biochanin A exhibited efflux pump inhibiting activity in *M. smegmatis* comparable to that of verapamil (VP) [22] and hence was used as template for synthesis of potent 3-phenylquinolone efflux inhibitors in *M. avium* [23]. Given the crucial problems posed by multidrug resistant pathogens, especially by mycobacteria, the administration of a plant-derived efflux pump inhibitor combined with an antibiotic may provide greater clinical benefits in the treatment of infectious diseases [24]. Flavonoids proved to be a promising group of plant phenolics in that respect and was hence investigated further in the present study by selecting more lipophilic structures, i.e., methoxylated derivatives (skullcapflavone II (**1**), nobiletin (**2**), tangeretin (**3**), wogonin (**5**)) and flavones lacking substituents at the C-2 aryl ring (baicalein (**4**), wogonin (**5**)) which might have a higher affinity for the lipid-rich mycobacterial cell envelope. Structures of the selected compounds can be depicted from Figure 1.

In this study, *M. smegmatis*, *M. aurum*, and *M. bovis* BCG were used as surrogate models for *M. tuberculosis* organism to analyze efflux-mediated resistance. We propose specific plant phenolic compounds, i.e., flavonoids, with strong antimycobacterial and resistance-modifying properties as valuable agents in the antibiotic therapy of mycobacterial infections. Additionally, we have demonstrated the ability of these phyto-compounds to impair the function of efflux pumps in mycobacteria. Two reference inhibitors, VP and chlorpromazine (CPZ), served to verify the efflux inhibiting profile of the suggested natural product compounds in the mycobacterial model strains.

## 2. Results

### 2.1. Antibacterial Activity

Five plant-derived flavonoids, skullcapflavone II (**1**), nobiletin (**2**), tangeretin (**3**), baicalein (**4**), and wogonin (**5**) were assessed for their antimicrobial activities against the non-pathogenic model organisms *M. smegmatis* mc^2^ 155, *M. aurum*, and *M. bovis* BCG. The minimum inhibitory concentrations (MICs) of the compounds as well as INH as reference antituberculotic against the studied strains are summarized in Table 1.

Briefly, the high-throughput spot culture growth inhibition (HT-SPOTi) assay [25,26] a rapid, gold standard, whole-cell screening method, was applied to investigate the anti-mycobacterial potential of the plant substrates against *M. aurum* and *M. bovis* BCG. The results indicate that all flavonoids exhibited strong antibacterial activities (MIC_99_ ≤ 31.25 mg/L) against *M. aurum*. Skullcapflavone II (**1**) was the most potent compound, with an MIC_99_ of 7.8 mg/L. However, baicalein (**4**) was the only compound showing strong antimycobacterial activity against *M. smegmatis* at a MIC_99_ of 32 mg/L.

### 2.2. Resistance Modulatory Activity

The resistance-modulating profile of the compounds towards *M. smegmatis*, a model strain that expresses a range of efflux pumps, was determined using a microtiter broth dilution assay. All compounds were tested at sub-inhibitory concentrations correlating to one-quarter of their MIC in *M. smegmatis*. The modulation factor (MF) indicates the resistance-modifying impact of the compounds on the MICs of ethidium bromide (EtBr) and rifampicin (RIF), a frontline antitubercular drug. Previous resistance modulation experiments in *M. smegmatis* with RIF showed better correlation to EtBr modulation than with INH [28], hence in the current study, RIF was preferred. Rodriguez et al. [29] tested RIF in combination with VP and CPZ in *M. smegmatis* mc^2^ 155 wild-type strain, which revealed a MF of 2 (VP) and 4 (CPZ), respectively. This also supported our selection of the *M. smegmatis* model and RIF as anti-tuberculotic drug in order to assay the flavonoids **1**-**5** for potential resistance modifying effects related to efflux pump inhibition. Although RIF resistance in *M. tuberculosis* strains mainly refers to chromosomal mutations, the overexpression of efflux pumps in a higher number of RIF-resistant strains compared to RIF-sensitive strains points towards a contribution of efflux to RIF resistance of *M. tuberculosis* [30].

Given the results of the modulatory screening presented in Table 2, it can be observed that the majority of the compounds affected the susceptibility of *M. smegmatis* towards EtBr and RIF, albeit to varying extents. Of all compounds tested, compound **1** proved to be the most effective modulator, causing a remarkable increase in the susceptibility of *M. smegmatis* to EtBr (MF = 128) and enhanced the activity of the anti-TB drug RIF to 4-fold.

### 2.3. EtBr Accumulation

Ethidium bromide, a known substrate of efflux pumps, is commonly used to assess efflux activity due to its ability to fluoresce when binding to hydrophobic regions within the bacterial cell [26]. The increase of EtBr-accumulation in the presence of a potential EPI indicates an interplay between cell-wall permeability and inhibition of efflux activity, which can be detected by fluorometric measurement [10,31].

Therefore, in order to assess the potential of the flavonoid compound group to inhibit mycobacterial efflux pumps, EtBr-accumulation assays were performed using the known EPIs VP and CPZ as reference inhibitors. Compounds were evaluated for their capability to accumulate EtBr in *M. smegmatis* and *M. aurum.* As shown in a previous study of Rodriguez et al. [27] in *M. bovis* BCG, only a basal efflux activity with EtBr took place, and the effects of EPIs in this strain were less clearly observable. This is also in agreement with our observations (unpublished data). Hence, in this study, we preferred to use *M. smegmatis* and *M. aurum* as model strains for EtBr accumulation in mycobacteria. Verapamil, a calcium-channel antagonist, clinically used for the treatment of cardiac disorders, is a well-known inhibitor of p-glycoprotein in mammalian cells [32] and was also found to inhibit ATP-dependent multidrug transporters (ABC pumps) in prokaryotic cells such as the DrrAB pump of *M. tuberculosis* [33,34]. VP and phenothiazines like CPZ are regarded as compounds, which interfere directly or indirectly with the proton motive force, the energy source of many secondary transporters [27].

Compounds were tested at concentrations half of their MIC together with 0.4% glucose and EtBr at 0.5 mg/L, the lowest concentration of EtBr that resulted in minimal accumulation by *M. smegmatis* and *M. aurum* within 60 min at 37 °C. The reference inhibitor VP indicated the highest level of EtBr–accumulation in both mycobacterial strains, while CPZ was less effective.

**Figure 2:**
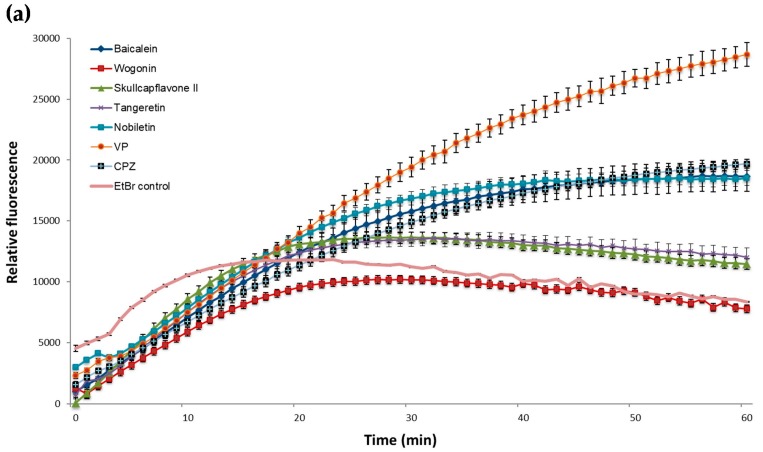

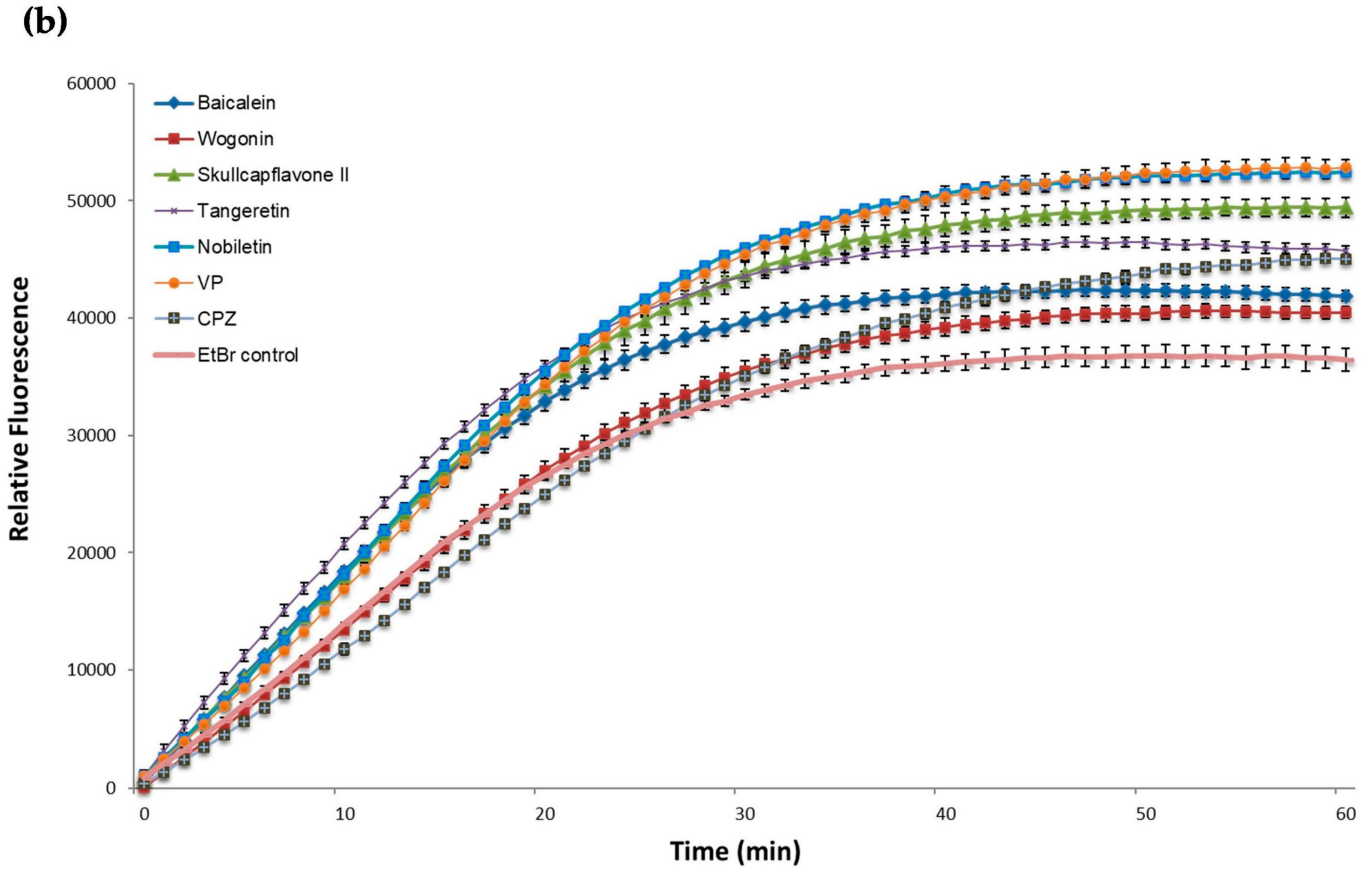
Effect of the potential EPIs and reference inhibitors on the EtBr accumulation in *M. smegmatis* mc^2^155 (**a**) and *M. aurum* ATCC 23366 (**b**). All compounds were tested at sub inhibitory concentrations at half of their MIC (Table 1). EtBr, ethidium bromide was used as negative control (drug-free culture) at a final concentration of 0.5 mg/L. VP, verapamil, a well-known efflux pump inhibitor and CPZ, chlorpromazine, were integrated as positive controls. The experiments were performed in triplicate (*n* = 3); values represent means + SD.

In EtBr accumulation assays with *M. smegmatis*, only flavonoids **2** and **4** caused an increase in accumulation similar to CPZ. As Figure 2a demonstrates, all other compounds could slightly potentiate accumulation when compared to the negative control but were less effective than the reference inhibitors.

Compound **2** was shown to be the most effective promoter for EtBr accumulation in *M. aurum* (Figure 2b), with a similar efficacy to that of VP. Compounds **1** and **3** enhanced EtBr-accumulation in *M. aurum* to a greater extent than CPZ but were less effective than VP. Flavonoids **4** and **5** were the least effective of the compounds tested against *M. aurum*.

## 3. Discussion

A small chemical series of plant-derived flavonoid compounds (**1**–**5**) was screened for antimycobacterial activity against a range of fast- and slow-growing mycobacterial model organisms. Efflux-pump inhibition and modulatory activity were tested against the fast-growing mycobacterial model organisms *M. smegmatis* and *M. aurum*, due to the limitations of the assay with regards to the slow-growing nature of *M. bovis* BCG.

Considering the structural similarities of the tested flavonoids, particular structural moieties in the molecule appear to be crucial for the antimicrobial properties. The antibacterial activity of flavonoids may be related to structural features such as the number and positions of methoxy and hydroxyl groups [35,36]. The 5,6,7-trihydroxy substitution pattern of compound **4** appears to enhance the antimycobacterial effect against *M. smegmatis*, as can be observed by comparison with the structurally similar flavonoid **5**. It is notable that the most potent inhibitor of *M. smegmatis* is the most hydrophilic of the five flavonoids tested. This may be influenced by the fact that, unlike the other mycobacterial strains, *M. smegmatis* possesses a higher number of porin-like channels such as the major porin MspA, which allows the diffusion of hydrophilic compounds through the hydrophobic bilayer of the cell wall [37,38]. On the other hand, the only flavonoid with the unusual 2′, 6′-substitution pattern, i.e., compound **1**, was the most active in *M. aurum*, and the more lipophilic polymethoxylated flavonoids **1**-**3** showed lower MIC values in *M. bovis* BCG compared to those compounds with no substituents at the C-2 aryl ring (compounds **4**, **5**), Table 1. In a previous study, it was reported that the presence of a lipophilic group at position 6 or 8 as well as hydroxylation at position 5 such as in compound **1** improves the antibacterial activity of flavonoids [39].

It is notable that across the three model organisms used in this study, the MIC_99_ values and relative potency of the five flavonoids shows significant variation. Such differences have been reported in other publications and may result from any of a number of phenotypic and genotypic variations between the three species [6,40]. For example, the number of unique proteins found in *M. smegmatis* was 224 greater than for *M. aurum*, *M. bovis* BCG, and *M. tuberculosis* [6].

Skullcapflavone II (**1**) was also identified as the most potent modulator of the antibacterial susceptibility of the compounds studied in *M. smegmatis*, with a MF of 128 when combined with EtBr and a MF of 4 when combined with RIF. It is important to note however, the results of the modulation assays simply indicate a possible trend towards efflux inhibition rather than a precise prediction [41,42]. An important role of efflux pumps, specifically LfrA, in resistance to *M. smegmatis* to RIF can be deduced from the results of Rodriguez et al. [29] who found a significant reduction of MIC for RIF when comparing susceptibility of wild-type strain (MIC = 4 mg/L) and a lfrA gene knockout strain (MIC = 0.5 mg/L) of *M. smegmatis* mc^2^ 155. However, in the current study, no specific mycobacterial efflux pump has been focused. Comparison of compound **1** and the similar flavonoids **2** and **3** suggests that the replacement of the C-5 methoxy group with a hydroxyl group may be significant. However, the substitution on the C-2 aryl ring may also have a significant influence of their EtBr- and RIF-modulating abilities. However, compounds **2** and **3**, both polymethoxyflavones distinguished by one methoxy- group at C-3′, exhibited similar modulating effects against the tested strains.

All compounds were observed to increase the level of EtBr-accumulation in *M. smegmatis* (with the exception of **5**) and *M. aurum* in relation to the negative control. Compound **2** in particular displayed potent efflux-inhibitory activity, and by comparison with the closely related flavonoid **3**, it can be observed that the additional 3′-methoxy group significantly improved the efflux inhibitory activity of this scaffold. Interestingly, flavonoid **4** caused a significant accumulation level for EtBr in *M. smegmatis* comparable to the effect of CPZ but had no modulatory activity on EtBr (MF = 1). On the other hand, compound **1**, which appeared to be a highly efficient modulator for EtBr against *M. smegmatis* (MF = 128), was comparatively less potent in terms of efflux inhibition. Consequently, a connection between strong modulatory and putative efflux activity of the compounds could not be observed. It has to be taken into consideration that due to the different exposure times of the bacteria to the test compounds and EtBr, i.e., the test substrate of potential efflux pumps, a comparison between both types of assay cannot readily be established. In the case of the modulation assay, incubation for 72 h takes place, whereas in the case of the accumulation assay, the mycobacteria are exposed for only 1 h to the respective chemicals. In addition, a high modulating activity in combination with EtBr, as in case of compound **1**, might influence the viability of the mycobacteria in the accumulation assay and finally lead to a less pronounced EtBr accumulation effect as one might expect from results of modulation assay. In previous experiments, we could show that VP and CPZ have a relatively low MF when combined with EtBr in *M. smegmatis* (VP, MF = 2 at 40 mg/L; CPZ, MF = 2 at 32 mg/L) [22], however both compounds clearly facilitated EtBr accumulation in the fluorescence-based accumulation assay. Cell wall impermeability in conjunction with the individual numbers of efflux pumps present in each mycobacterial strain may contribute to the diversity of drug susceptibility between the model strains. The overactivity of efflux systems as well as the mode of inhibition is still mostly unexplored [43].

The results of this study can be taken as a starting point for more in depth investigation of skullcapflavone II (**1**) and nobiletin (**2**) as adjuvants in antimycobacterial combination therapy. Several polymethoxylated flavones, bearing four or more methoxyl groups on their benzo-γ-pyrone skeleton, have previously been shown to possess a broad spectrum of biological activities, including anti-inflammatory, anti-carcinogenic, antiviral, antioxidant, anti-thrombogenic, and anti-atherogenic properties [39,44].

## 4. Materials and Methods

### 4.1. Drugs and Inhibitors

Plant compounds skullcapflavone II (**1**), nobiletin (**2**), and tangeretin (**3**) were purchased from ChemFaces Biochemical Co., Wuhan, China. Baicalein (**4**) and wogonin (**5**) were obtained from Sigma Aldrich. Purities of the compounds were ≥98%. Chlorpromazine, verapamil, rifampicin, ethidium bromide, isoniazid, and phosphate-buffered saline (PBS) in tablets (0.01 M phosphate buffer, 0.0027 M KCl, 0.14 M NaCl and 0,05% Tween 80, pH = 7.4) were purchased from Sigma Aldrich (Poole, UK).

All compounds were dissolved either in dimethyl sulfoxide (DMSO) or in sterile deionized water.

### 4.2. Bacterial Strains and Growth Media

The bacterial strains *Mycobacterium smegmatis* mc^2^ 155 ATCC 700084, *Mycobacterium aurum* ATCC 23366, and *Mycobacterium bovis* BCG ATCC 35734 were cultivated either on Columbia blood agar supplemented with 5% defibrinated horse blood (MIC and modulation assay) or in Middlebrook 7H9 broth supplemented with 10% of OADC enrichment (SPOTi and ethidium bromide accumulation assay) at 37 °C under aerobic conditions. Bacterial strains were purchased from UK National Collection of Type Cultures (NCTC). Middlebrook 7H9 broth supplemented with Middlebrook OADC Enrichment and 0.05% Tween 80 (Sigma-Aldrich) or 0.4% glycerol used for efflux assays were purchased from Becton–Dickinson (Oxford, UK). Mueller–Hinton Broth (cation-adjusted) was used for MIC and modulation experiments.

### 4.3. Drug Susceptibility Testing

Minimum inhibitory concentrations (MIC) of the compounds against non-pathogenic strains *Mycobacterium aurum* and *Mycobacterium bovis* BCG were determined by using the spot-culture growth inhibition assay (SPOTi) according to Evangelopoulos and Bhakta [25].

Isoniazid, a first-line antitubercular drug, was included as a positive control. All experiments were performed in triplicate. The MIC, defined as the lowest concentration of a given compound required to inhibit bacterial growth, was visually determined.

Microbroth dilution assays served to investigate the antimicrobial and resistance-modulating effects of the compounds against *Mycobacterium smegmatis* mc^2^ 155 (ATCC 700084) and were conducted as described in literature in accordance with NCCLS guidelines [43].

Briefly, all compounds were dissolved in dimethyl sulfoxide (DMSO) and diluted in Mueller–Hinton broth before testing in 96-well microtiter plates. The concentration of DMSO did not exceed 3.6% for the final assay. For each plate, an additional sterile and growth control as well as a positive control (Isoniazid) was included. The plates were inoculated with a 5 × 10^5^ colony forming units (CFU)/mL bacterial suspension, adjusted to equal the McFarland 0.5 turbidity standard, and were incubated at 37 °C for 72 h. For the assessment of cell viability, the determination of the MIC was carried out using MTT (3-(4,5-dimethylthiazol-2-yl)-2,5-diphenyltetrazolium bromide (Sigma-Aldrich), indicating bacterial growth by a visible color change from yellow to blue [45].

### 4.4. Modulation Factor Analysis

Additionally, test compounds were further analyzed for their modulatory activity on the MIC of ethidium bromide (EtBr) and rifampicin (RIF), respectively.

At sub-inhibitory concentrations corresponding to one-quarter of their MIC, the concentration of the compounds was kept constant while EtBr and RIF were serially diluted. The modulation factor (MF) reflected the modulating impact of the compounds on the MIC of the antibiotic and ethidium bromide, calculated according to the formula:

MF = (MIC antibiotic)/(MIC antibiotic + modulator)(1)

### 4.5. Whole Cell Efflux Assay

EtBr accumulation assay for the bacterial strains *Mycobacterium smegmatis mc^2^*155 and *Mycobacterium aurum* was adapted following the procedure of Rodrigues et al. [7]. Each compound was tested at sub inhibitory concentrations correlating to one-half of their MIC.

Bacterial cultures were grown in 10 mL of Middlebrook 7H9 broth medium supplemented with OADC at 37 °C until an OD_600_ of 0.8 was reached. After adjusting the OD_600_ to 0.4 in Middlebrook 7H9 broth, the bacterial suspension was centrifuged at 3000 rpm for 10 min. The supernatant was discarded, and the pellet re-suspended in 10 mL of PBS containing 0.05% Tween 80. Test compounds at sub-inhibitory concentrations (MIC 1/2) together with glucose (final concentration of 0.4%) were prepared in microtubes containing 0.5 mL of bacterial suspension. Aliquots of 0.1 mL of each working solution were transferred into separate wells of a 96-well plate. Ethidium bromide was added to each well to a final concentration of 0.5 mg/L.

The 96-well plate was placed in a fluorimeter (FLUOstar OPTIMA, BMG Labtech) and fluorescence data were recorded every 60 s for 60 min at 37 °C using an excitation wavelength of 544 nm and an emission wavelength of 590 nm. The effect of the reference inhibitors VP and CPZ on the accumulation of EtBr at 37 °C was determined as described before. All experiments were performed in triplicate.

In order to evaluate the homogeneity of variance and statistical significance of the efflux results, the f-test and two-tailed t-test were conducted to allow comparison of the test compounds to the EtBr control (Table 3).

## 5. Conclusions

The polymethoxylated flavonoids skullcapflavone II (**1**) and nobiletin (**2**) were found to be potent inhibitors of the growth of *M. aurum* and *M. bovis* BCG and interfered with efflux pump activity in *M. aurum* and *M. smegmatis*. In particular, flavonoid **1** exerted a remarkable modulating impact on EtBr and significantly decreased the rifampicin-resistance level of *M. smegmatis*. These results are significant as several reports have shown that efflux pump inhibitors are able to inhibit the survival of intracellular *M. tuberculosis* and potentiate the activity of anti-TB drugs [46,47]. However, it was not possible to observe a clear connection between strong modulatory and putative efflux activity of the compounds.

To date, there have been no published studies into the antimycobacterial efficacy of skullcapflavone II (**1**) and nobiletin (**2**) or their inhibitory activity on drug efflux in mycobacteria. It is imperative that we explore new strategies in TB treatment and management, and therefore we propose these flavonoids are valuable candidates for the inhibition of mycobacterial efflux pumps and the improvement of antibiotic performance against tuberculosis and other non-tubercular mycobacterial infections.

## Figures and Tables

**Table 1:** Minimum inhibitory concentrations (MICs) of ethidium bromide and efflux pump inhibitors (EPIs) determined for *M. smegmatis* mc^2^ 155 using microbroth dilution assay and spot culture growth inhibition (SPOTi)-assay for *M. aurum* and *M. bovis* BCG.

	MIC_99_ (mg/L)		
Compound	*M. smegmatis* mc^2^ 155	*M. aurum* ATCC 23366	*M. bovis* BCG ATCC 35734
Skullcapflavone II (**1**)	128	7.8	31.25
Nobiletin (**2**)	128	31.25	62.5
Tangeretin (**3**)	128	31.25	31.25
Baicalein (**4**)	32	31.25	250
Wogonin (**5**)	128	31.25	500
Isoniazid (INH)	4	0.5	0.1
Verapamil (VP)	250	250	320 [27]
Chlorpromazine (CPZ)	25	20	20 [27]
Ethidium bromide (EtBr)	6.25	1	0.5 [27]

**Table 2:** Determination of the MICs and modulation factors of the compounds for *M. smegmatis* mc^2^ 155 by using microbroth dilution assays.

Compound	MIC (mg/L)	[c] as Modulator (mg/L)	MIC Mod. (mg/L)/MF EtBr	MIC Mod. (mg/L)/MF RIF
Skullcapflavone II (**1**)	128	32	0.0625/128	8/4
Nobiletin (**2**)	128	32	2/4	32/1
Tangeretin (**3**)	128	32	2/4	32/1
Baicalein (**4**)	32	8	8/1	32/1
Wogonin (**5**)	128	32	4/2	16/2

Minimum inhibitory concentration (MIC) of ethidium bromide (EtBr) = 8 mg/L and MIC of rifampicin (RIF) = 32 mg/L. Modulation factor (MF) = MIC (EtBr or RIF)/MIC (EtBr or RIF + modulator); *n* = 4. MIC mod. = MIC of EtBr or RIF in presence of the modulator at a concentration corresponding to a quarter of its MIC.

**Table 3:** Results of the relative fluorescence of accumulation.

	*M. smegmatis* mc^2^ 155		*M. aurum* ATCC 23366	
Compound	Mean Value	Standard Deviation	Mean Value	Standard Deviation
Baicalein	18573,3 ***	102,1	42146,5 ***	151,3
Wogonin	8376,9 **	326,1	40517,0 ***	63,1
Skullcapflavone II	11811,1 ***	239,6	49361,2 ***	82,5
Tangeretin	12400,6 ***	202,7	46077,8 ***	213,4
Nobiletin	18451,2 ***	33,8	52316,3 ***	117,2
EtBr control	8778,6	223,8	36683,7	93,2
	*n* = 60		*n* = 60	

Calculated as measured during the last 10 min of the assays. Compounds were compared to the EtBr control. Asterisks indicate the level of significance: *** *p* < 0.001, ** *p* < 0.01.
